# Accuracy of dual‐energy computed tomography for the quantification of iodine in a soft tissue‐mimicking phantom

**DOI:** 10.1120/jacmp.v16i5.5519

**Published:** 2015-09-08

**Authors:** Jung‐Hui Li, Yeh‐Ming Du, Hsuan‐Ming Huang

**Affiliations:** ^1^ Department of Diagnostic Radiology Chang Gung Memorial Hospital, Kaohsiung Medical Center Kaohsiung Taiwan; ^2^ Department of Diagnostic Radiology Lotung Poh‐Ai Hospital Yilan County Taiwan; ^3^ Medical Physics Research Center Institute of Radiological Research, Chang Gung University and Chang Gung Memorial Hospital Taoyuan Taiwan

**Keywords:** dual‐energy CT, dual‐source CT, integrated detector, iodine quantification

## Abstract

The objective of this study was to evaluate the accuracy of dual‐energy CT (DECT) for quantifying iodine using a soft tissue‐mimicking phantom across various DECT acquisition parameters and dual‐source CT (DSCT) scanners. A phantom was constructed with plastic tubes containing soft tissue‐mimicking materials with known iodine concentrations (0–20 mg/mL). Experiments were performed on two DSCT scanners, one equipped with an integrated detector and the other with a conventional detector. DECT data were acquired using two DE modes (80 kV/Sn140 kV and 100 kV/Sn140 kV) with four pitch values (0.6, 0.8, 1.0, and 1.2). Images were reconstructed using a soft tissue kernel with and without beam hardening correction (BHC) for iodine. Using the dedicated DE software, iodine concentrations were measured and compared to true concentrations. We also investigated the effect of reducing gantry rotation time on the DECT‐based iodine measurement. At iodine concentrations higher than 10 mg/mL, the relative error in measured iodine concentration increased slightly. This error can be decreased by using the kernel with BHC, compared with the kernel without BHC. Both 80 kV/Sn140 kV and 100 kV/Sn140 kV modes could provide accurate quantification of iodine content. Increasing pitch value or reducing gantry rotation time had only a minor impact on the DECT‐based iodine measurement. The DSCT scanner, equipped with the new integrated detector, showed more accurate iodine quantification for all iodine concentrations higher than 10 mg/mL. An accurate quantification of iodine can be obtained using the second‐generation DSCT scanner in various DE modes with pitch values up to 1.2 and gantry rotation time down to 0.28 s. For iodine concentrations ≥10 mg/mL, using the new integrated detector and the kernel with BHC can improve the accuracy of DECT‐based iodine measurements.

PACS number: 87.57.Q‐

## I. INTRODUCTION

With the introduction of the first commercial dual‐source CT (DSCT) scanner, dual‐energy CT (DECT) has brought upon a number of clinical applications such as material differentiation and tissue characterization.^1^, ^2^ Among these applications, iodine extraction using DECT has been shown to be advantageous for many clinical studies.^3^, ^4^, ^5^, ^6^ For example, an iodine distribution map derived from DECT can be used to evaluate myocardial perfusion defects instead of high‐dose dynamic myocardial perfusion CT imaging.^(3)^ This iodine map can also be used for the detection of perfusion defects in cases of pulmonary embolism, as well as the characterization of pulmonary nodules in cases of suspected lung cancer.^(4)^ More importantly, iodine distribution map that combines with virtual noncontrast (VNC) and monoenergetic images can further contribute to lesion detection and tissue characterization.^5^, ^6^

The common method of diagnosis is the subjective visual evaluation of a DECT‐derived iodine map. In addition, recent studies reported that quantifying iodine concentration has the potential to distinguish different types of tumors^7^, ^8^ and to monitor therapy response.^9^, ^10^ However, a number of CT artifacts, such as beam hardening, partial volume averaging and motion misregistration, may cause artifactual perfusion defects^(4)^ and quantitative errors^11^, ^12^ in the DECT data analysis. Before evaluating the clinical value of DECT‐based iodine quantification, the acquisition and reconstruction parameters optimized for iodine extraction should be investigated.

To our knowledge, few studies have been conducted to examine the accuracy of DECT‐derived iodine concentration.^13^, ^14^, ^15^ Effects of DE acquisition mode, pitch, gantry rotation time, and reconstruction kernel on the accuracy of DECT‐based iodine quantification have not been investigated. In addition, it remains unclear whether the new second‐generation DSCT scanner equipped with an integrated circuit detector can provide more accurate iodine quantification. Therefore, the aim of this study was to assess the accuracy of DECT‐derived iodine concentration in a soft tissue‐mimicking phantom using various DE acquisition parameters and two commercial DSCT scanners, one equipped with an integrated circuit detector and the other with a conventional detector.

## II. MATERIALS AND METHODS

### A. Phantom setup

In this study, we made a soft tissue‐mimicking phantom which simulated contrast enhancement in normal liver tissue. To mimic normal liver tissue, we used a 18.5% w/v (weight/volume) glucose solution which has almost identical CT attenuation values of normal liver.^16^, ^17^ The iodine contrast medium (Omnipaque 350, GE Healthcare, Cork, Ireland) was added to the glucose solution with appropriate volume to the final concentration of 0, 2.5, 5, 10, 15, and 20 mg iodine per mL. Each solution was sealed in a 320 mL plastic tube which had a diameter of 5.3 cm and a height of 14.5 cm. Six tubes were placed in a grid and submerged in a body‐sized plastic tank (length×width×height:28 cm×20 cm×28 cm) filled with water.

### B. DECT data acquisition and reconstruction

Experiments were performed with two second‐generation DSCT scanners (Somatom Definition Flash, Siemens Healthcare, Forchheim, Germany), one equipped with a conventional detector and the other with an integrated circuit detector (Stellar Detector; Siemens Healthcare). These two types of detectors used distributed and integrated electronics, respectively. For each DSCT scanner, the soft tissue‐mimicking phantom was scanned using two DE acquisition modes (80 kV/Sn140 kV and 100 kV/Sn140 kV) and four pitch values (0.6, 0.8, 1.0, and 1.2). The other scanning parameters were as follows: detector of 32 collimated 0.6 mm rows and gantry rotation time of 0.5 s. For each DE scan, automatic tube current modulation was used. Table 1 summarizes the CT dose index volume (${\text{CTDI}}_{\text{vol}}$) and effective mAs of each DE scan. All images were reconstructed at a slice thickness of 1.5 mm and an increment of 1 mm, with a 512×512 matrix using the conventional filtered back projection (FBP) algorithm with two different kernels: a medium smooth kernel (D30f) and a medium smooth kernel with beam‐hardening correction (BHC) for iodine (D33f).

**Table 1:** CT dose index volume (${\text{CTDI}}_{\text{vol}}$) and effective mAs of each dual‐energy scan

*DE mode*		*80 kV/Sn140 kV*				*100 kV/Sn140 kV*			
	*Pitch*	*0.6*	*0.8*	*1*	*1.2*	*0.6*	*0.8*	*1*	*1.2*
Conventional detector	CTDI_VOL_ (mGy)	10.51	10.83	10.62	9.84	10.89	10.90	10.85	10.86
	Effective mAs	270/111	283/111	278/111	241/111	137/111	136/111	137/111	137/111
Integrated detector	CTDI_VOL_ (mGy)	7.28	7.27	7.55	7.52	8.05	8.10	8.11	8.21
	Effective mAs	176/83	175/83	188/84	184/85	100/83	101/83	102/84	103/84

To further understand the performance of DECT‐based iodine quantification, we conducted two additional studies using the DSCT scanner with a conventional detector. First, each DE scan was repeated three times to test the reproducibility of DECT‐based iodine quantification. Second, we investigated the effect of gantry rotation time on the iodine concentration measurements. The phantom was scanned at gantry rotation time of 0.50 s, 0.33 s, and 0.28 s. For each gantry rotation time, the scanning parameters were as follows: detector collimation of 32×0.6 mm, two DE acquisition modes (80 kV/Sn140 kV and 100 kV/Sn140 kV), and two pitch values (0.6 and 1.2). All images were reconstructed at a slice thickness of 1.5 mm and an increment of 1 mm, with a 512×512 matrix using the conventional FBP algorithm with a D30f kernel.

### C. DECT data postprocessing and analysis

**Figure 1:**
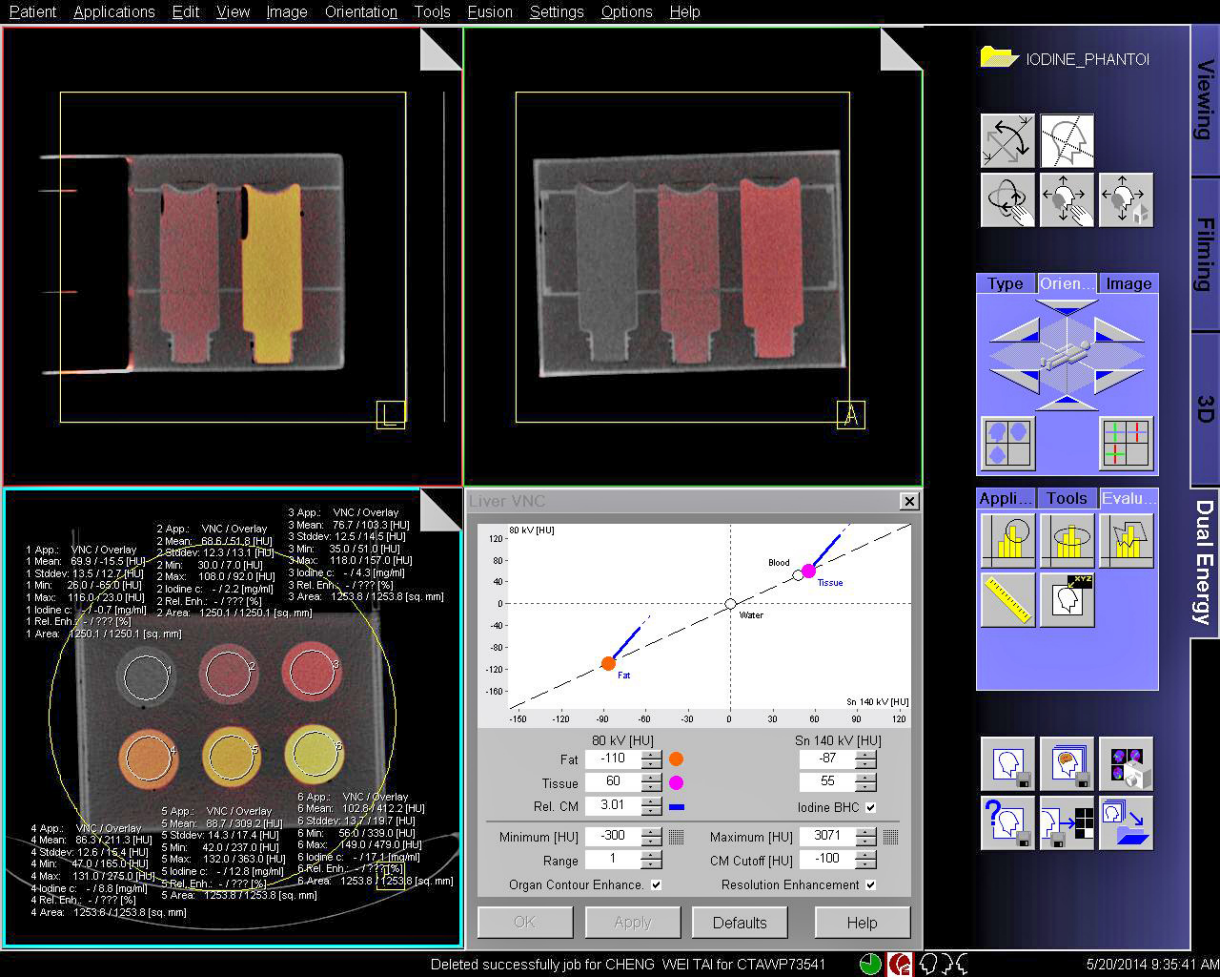
Screenshot of the Liver VNC application used for quantifying the iodine concentration. Images in the panel are fusion images of color‐coded iodine images and gray‐scale virtual noncontrast images.

Each DE image set (low‐ and high‐kV pair) was transferred to a workstation (Syngo Multi‐Modality Workplace [MMWP], Siemens Healthcare) for quantifying iodine concentration using the Liver VNC DE application class (Syngo, Dual‐energy, MMWP, version VE 50A). With the assumption that three basic materials were fat, soft tissue, and iodine, the three‐material decomposition algorithm was used to extract iodine content from contrast‐enhanced DECT images.^(2)^ Color‐coded iodine images were superimposed on VNC images for visualization of iodine distribution and anatomic information simultaneously (Fig. 1).^(18)^

In this study, we used the default settings of Liver VNC (Fig. 1). For the 80 kV/Sn140 kV DE acquisition mode, the nominal CT numbers of fat and soft tissue at low (high) tube voltage were −110 (87) HU and 60 (55) HU, respectively. For the 100 kV/Sn140 kV DE acquisition mode, the nominal CT numbers of fat and soft tissue at low (high) tube voltage were −103 (87) HU and 57 (55) HU, respectively. The relative contrast medium (Rel. CM) enhancement, which is the ratio of the iodine enhancement at low kV and that at high kV, was 3.01 and 2.24 for 80 kV/Sn140 kV and 100 kV/Sn140 kV acquisition modes, respectively. The minimum cutoff was −300 HU and the maximum cutoff was 3071 HU. According to the mixed image (i.e., a weighted average image obtained from the low‐ and high‐kV images), pixels with values below the minimum cutoff value or above the maximum cutoff value were set to zero in the iodine image. In the iodine image, pixels below the value of CM Cutoff (i.e., −100 HU) were set to zero. The range parameter that controls spatial resolution of the VNC image was set to 1. The default settings for iodine BHC, resolution enhancement, and organ contour enhancement were used here.

A circular region of interest (12.5 cm^2^ in area) was placed centrally in each of the six tubes to measure the iodine concentration (Fig. 1). For each DSCT scanner, 96 measurements were performed to measure six iodine concentrations at eight protocol combinations (two DE acquisition modes with four different pitch values) and two reconstruction kernels. The relative difference (=(measured–true)/true×100) was used to evaluate the accuracy of measured iodine quantification.

Using the same procedure as mentioned above, 72 measurements were performed to measure six iodine concentrations at twelve protocol combinations (two DE acquisition modes, two pitch values, and three gantry rotation times). To assess the effect of reducing gantry rotation time on the iodine concentration measurements, the relative difference between measured iodine concentrations obtained with the gantry rotation time of 0.5 s and the shorter gantry rotation times (i.e., 0.33 s and 0.28 s) was calculated.

## III. RESULTS

### A. DSCT scanner equipped with a conventional detector

**Figure 2:**
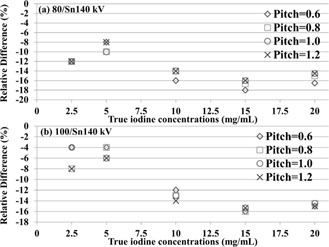
Relative differences (%) between true and measured iodine concentrations with DE scans operated at (a) 80 kV/Sn140 kV and (b) 100 kV/Sn140 kV modes.

We observed that measured iodine concentration obtained from DECT (using the D30f kernel) with either 80 kV/Sn140 kV configuration (Fig. 2(a)) or 100 kV/Sn140 kV configuration (Fig. 2(b)) was almost independent of the pitch value. As the concentration of iodine was greater than or equal to 10 mg/mL, the relative error in iodine concentration measurement increased slightly. In addition, we found that DECT‐based iodine quantification systematically underestimated concentrations in all concentration ranges.

**Figure 3:**
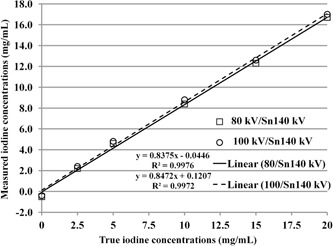
Scatterplot and linear regression for correlation between true and measured iodine concentrations with 80 kV/Sn140 kV and 100 kV/Sn140 kV modes at pitch=0.6. The coefficient of determination ($R^{2}$) is a measure of goodness of fit.

Figure 3 shows an example of the scatter plot of true iodine concentrations versus DECT‐derived iodine concentrations measured using 80 kV/Sn140 kV and 100 kV/Sn140 kV modes at pitch=0.6. Linear regression yielded excellent agreement ($R^{2}>0.99$) between true and measured iodine concentrations, indicating that the DECT‐based measurement could reveal actual changes in iodine concentrations.

**Figure 4:**
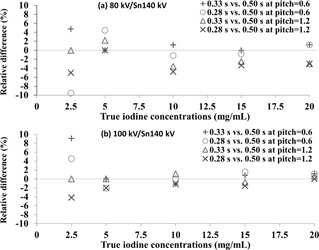
Relative differences (%) in iodine concentrations between the gantry rotation time of 0.5 s and the shorter gantry rotation times (i.e., 0.33 s and 0.28 s) operated at (a) 80 kV/Sn140 kV and (b) 100 kV/Sn140 kV modes at pitch=0.6 and 1.2.

The reproducibility test showed that the difference in iodine concentrations between repeated measurements was within ±0.2 mg/mL. This finding demonstrated that the second‐generation DSCT scanner equipped with a conventional detector can provide a reproducible measurement of iodine concentrations. Based on the image datasets reconstructed using the D30f kernel, the relative difference between measured iodine concentrations obtained with the gantry rotation time of 0.5 s and those obtained with the shorter gantry rotation times (i.e., 0.33 s and 0.28 s) is shown in Fig. 4 for (a) 80 kV/Sn140 kV and (b) 100 kV/Sn140 kV modes with pitch=0.6 and 1.2. The relative difference of iodine concentrations was within ±10%.

Table 2 illustrates measured iodine concentrations obtained from 80 kV/Sn140 kV and 100 kV/Sn140 kV modes at pitch=0.6 and 1.2. Both 80 kV/Sn140 kV and 100 kV/Sn140 kV modes had similar accuracy in quantifying iodine concentrations. In addition, Table 2 shows the comparison of DECT‐based iodine measurements obtained using two different reconstruction kernels (D30f and D33f). For iodine concentrations higher than 2.5 mg/mL, the error in iodine concentration measurement decreased using the D33f kernel compared to the D30f kernel. However, for the iodine concentration of 2.5 mg/mL, the D33f kernel showed more underestimation than the D30f kernel. The same results could be obtained from all other values of pitch.

**Table 2:** Iodine concentrations measured with the dual‐source CT scanner equipped with a conventional detector

	*Measured Iodine Concentration (mg/mL)*							
*True Iodine Concentration (mg/mL)*	*D30f Kernel*				*D33f Kernel*			
	*80 kV/Sn140 kV*		*100 kV/Sn140 kV*		*80 kV/Sn140 kV*		*100 kV/Sn140 kV*	
	Pitch=0.6	Pitch=1.2	Pitch=0.6	Pitch=1.2	Pitch=0.6	Pitch=1.2	Pitch=0.6	Pitch=1.2
0	−0.5	−0.6	−0.4	−0.5	−0.5	−0.6	−0.5	−0.5
2.5	2.2	2.2	2.4	2.3	2.0	2.0	1.8	1.9
5.0	4.6	4.6	4.8	4.7	5.1	5.1	5.0	4.9
10	8.4	8.6	8.8	8.6	9.2	9.4	9.6	9.5
15	12.3	12.6	12.6	12.7	13.8	14.2	14.1	14.1
20	16.7	17.1	17.0	17.0	18.4	18.9	18.5	18.3

### B. DSCT scanner equipped with an integrated circuit detector

Overall, we observed the same effects of using various DE modes, pitch values, and reconstruction kernels on iodine quantification in the DSCT scanner equipped with an integrated circuit detector as compared to the same scanner equipped with a conventional detector. Table 3 shows iodine concentrations measured with two DSCT scanners, one equipped with a conventional detector and the other with an integrated circuit detector, at pitch=0.6. The accuracy of quantifying iodine concentrations of 0–5 mg/mL for the two DSCT scanners was similar. However, for iodine concentrations higher than (or equal to) 10 mg/mL, the use of an integrated circuit detector led to more accurate iodine quantification, compared with the use of a conventional detector. Moreover, using the kernel with BHC (D33f) and an integrated circuit detector yielded minimum errors in measurements of high iodine concentrations (higher than or equal to 10 mg/mL). Results obtained from all other values of pitch showed the same findings.

**Table 3:** Iodine concentrations measured with two dual‐source CT scanners, one equipped with a conventional detector and the other with an integrated detector, at pitch=0.6

	*Measured Iodine Concentration (mg/mL)*							
*True Iodine Concentration (mg/mL)*	*D30 Kernel*				*D33 Kernel*			
	*80 kV/Sn140 kV*		*100 kV/Sn140 kV*		*80 kV/Sn140 kV*		*100 kV/Sn140 kV*	
	*Conventional Detector*	*Integrated Detector*	*Conventional Detector*	*Integrated Detector*	*Conventional Detector*	*Integrated Detector*	*Conventional Detector*	*Integrated Detector*
0	−0.5	−0.7	−0.4	−0.6	−0.5	−0.7	−0.5	−0.6
2.5	2.2	2.2	2.4	2.5	2.0	2.0	1.8	2.1
5.0	4.6	4.4	4.8	4.7	5.1	4.8	5.0	5.2
10	8.4	8.8	8.8	9.0	9.2	9.6	9.6	10.1
15	12.3	12.8	12.6	13.1	13.8	14.3	14.1	15.2
20	16.7	17.0	17.0	17.3	18.4	18.9	18.5	19.5

## IV. DISCUSSION

In this study, we used a soft tissue‐mimicking phantom to assess the effects of DE acquisition mode, pitch value, gantry rotation time, reconstruction kernel, and integrated circuit detector on the accuracy of DECT‐based iodine quantification. For iodine concentrations in the range of 0–20 mg/mL,^(15)^ DECT with various acquisition and reconstruction parameters can provide promising accuracy for quantifying iodine concentration.

To simulate contrast enhancement in normal soft tissue such as liver, iodine contrast medium was diluted with a liver‐mimicking material (18.5% w/v glucose solution).^16^, ^17^ This is different from the previous studies that used solutions prepared by diluting iodine contrast medium with water or normal saline solution.^13^, ^14^, ^15^ Our purpose is to mimic the contrast enhancement in soft tissue. However, unlike the semianthropomorphic liver phantom (QRM, Germany) that simulates anatomical structures of the abdomen, the phantom used in this study may be too simple to simulate a real patient. Further validation of DECT‐based iodine quantification using a semianthropomorphic liver phantom is required.

As compared to true iodine concentrations, all measured iodine concentrations were underestimated (Fig. 2). It seems that higher iodine content (≥10 mg/mL) may lead to severe underestimation of iodine concentrations (12%–18%). This may be caused by beam‐hardening artifacts, as reported in some previous studies.^12^, ^19^ Thus, images were reconstructed using the kernel with BHC (D33f). In comparison with the kernel without BHC (D30f), the kernel with BHC (D33f) showed a 4%–8% underestimation for the region with high iodine concentrations (≥10 mg/mL). However, for the iodine concentration of 2.5 mg/mL, the D33f kernel led to more underestimation, compared to the D30f kernel. Since the region with the iodine concentration of 2.5 mg/mL was not affected by beam hardening, using the BHC reconstruction kernel (i.e., D33f) might introduce bias into the calculation of iodine concentration.

**Figure 5:**
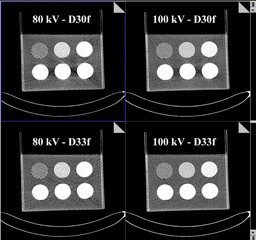
An example of 80 kV and 100 kV images reconstructed using D30f and D33f kernels.

In this study, three tubes with higher iodine concentrations were placed in the same row. As a result, beam‐hardening artifacts which took place in these three tubes (Fig. 5) may affect each other's iodine measurements. Thus, for the three inserts with high iodine concentrations, we scanned one at a time. With results obtained using the D30f kernel (Table 2), we found that the simultaneous scanning of three inserts led to a 12%–18% underestimation, whereas the individual scanning (i.e., scan one insert at a time) showed a 4%–8% underestimation. This finding indicates that for each of the three inserts, there is an 8%–10% underestimation resulting from the other two inserts. Furthermore, the 4%–8% underestimation could be reduced by using the BHC kernel.

We found that DECT‐based iodine quantification obtained using different values of pitch (0.6, 0.8, 1.0, and 1.2) showed almost the same accuracy (Fig. 2). This finding suggests that the spiral DE scan mode with pitch values of up to 1.2 may be performed in clinical studies. The advantage of using high‐pitch DECT scan is the reduction of scan time, which can be beneficial for patients with limited breath‐holding capability. More importantly, using various values of pitch had no significant impact on radiation dose (Table 1). Future studies using patient data are necessary to further validate the results of our study.

We demonstrated that both 80 kV/Sn140 kV and 100 kV/Sn140 kV acquisition modes had similar accuracy for quantifying iodine concentration (Table 2). It may be due to the fact that the effective mAs value of 80 kV was 1.7 to 2 times higher than that of 100 kV (Table 1). The increase of the tube current product improved the image quality of 80 kV. For large patient sizes, the low tube voltage should be set to 100 kV if automatic tube current modulation was not used. In this study, we didn't vary phantom size due to the results reported by previous phantom studies^13^, ^15^ which have shown that the phantom size has no significant effect on the measured iodine concentration using the DSCT scanner.

One previous study reported that, in a single‐source DECT scanner with fast kilovoltage switching, there was a larger quantification error when the gantry rotation time was reduced.^(20)^ Thus, we investigated the effect of reducing gantry rotation time on the accuracy of iodine quantification in the second‐generation DSCT scanner. As shown in Fig. 4, we found that reducing gantry rotation times led to small changes (±10%) in iodine concentration measurements in all conditions. This finding suggests that the spiral DE scan mode with shorter gantry rotation time can provide accurate iodine measurement. One potential benefit of reducing gantry rotation time is the reduction of scan time, which may be useful for patients with poor breath‐holding capability.

As compared to the DSCT scanner equipped with a conventional detector, the same scanner equipped with an integrated circuit detector showed no improvement in iodine quantification for iodine concentrations ranged from 0 to 5 mg/mL. However, for iodine concentrations ranged from 10 to 20 mg/mL, the use of an integrated circuit detector could improve the accuracy of DECT‐based iodine quantification compared with the use of a conventional detector. It is well known that beam‐hardening artifacts, as well as streak artifacts, exist in low‐kV images through highly attenuating materials such as high concentrations of iodine. As a result, the integrated circuit detector that reduces electronic noise and streak artifacts^(21)^ may lead to a more accurate quantification of high iodine concentrations.

Overall, radiation dose was 20%–30% less for the DSCT scanner equipped with an integrated circuit detector, as compared to the same scanner equipped with a conventional detector (Table 1). It should be noted that both DSCT scanners have the same exposure control settings. This finding demonstrated that the use of an integrated circuit detector has the potential to perform a lower‐dose DECT scan while still providing accurate iodine quantification. In addition to the use of integrated circuit detectors, the use of iterative reconstruction (IR) algorithms may be a good strategy for further reducing the radiation dose.^(22)^ Future studies are required to validate whether an IR algorithm can reduce the radiation dose of a DECT scan while maintaining the accuracy of DECT‐based iodine quantification.

## V. CONCLUSIONS

The second‐generation DSCT scanner can provide accurate and reproducible measurements of iodine concentration using various DE acquisition modes, pitch values up to 1.2, and gantry rotation time down to 0.28 s. For iodine concentrations beyond the physiologic range (higher than or equal to 10 mg/mL), the use of an integrated circuit CT detector and the kernel with BHC can improve the accuracy of DECT‐based iodine quantification.
